# Supplementary material for: Migration and interbirth transition rate using Benin Demographic and Health Survey data: Does episode-splitting matter?
Source: PLoS One. 2021 Oct 22;16(10):e0258297. doi: 10.1371/journal.pone.0258297 (PMC8535178; doi:10.1371/journal.pone.0258297)
Supplement: Attachment — Submitted filename: PONE-D-2120332.docx [file pone.0258297.s001.docx]

Peer review: PONE-D-21-20332

Introduction

This article provides an interesting and detailed explanation of how episode splitting in migration studies affects estimates of inter-birth intervals, and the implications of this for our understanding of acclimatization of migrants to new settings. The methods are correct and the study conclusions justified from the results and methods. I have no significant concerns with the paper, and just a few comments which I hope the authors will address.

**Comments**

*Comparison with standard time-varying covariates*: It is not clear to me that the method employed here is different to a standard survival analysis with time-varying covariates, usually implemented in Stata using the stsplit command. Is the issue here that by using a directly-defined likelihood it is possible to adjust for survey sampling effects? Is the specific definition of an exponential distribution for the likelihood somehow unusual? I think the authors need to specify in more detail what new material their method adds. IN particular, if they have a specifically defined likelihood, how did they incorporate the survey sample process into that Likelihood? This is not specified in the methods. I think they just ran stsplit and then conducted survival analysis with a time-varying covariate? A little more detail about this in the methods would help to clarify the novelty of the study.

*Adjustment for wealth*: I assume that the Benin DHS includes a variable for wealth quintile, and I wonder whether the effect of migration persists after adjusting for wealth quintile. Does wealth quintile change after migration? Were the migrant women more likely to be of a lower or high wealth quintile, with different birth intervals? The authors have adjusted for education but not for wealth quintile, which is standard practice in this kind of study. I think they should justify this decision or add this covariate to their model (sorry in advance if it makes the results non-significant; I still think the paper is publishable if a non-significant result arises, though obviously the discussion would need to change a lot).

*Importance of the effect*: In the basic model (not incorporating migration timing) the hazard ratios are quite small. What is the public health and demographic significance of these small hazard ratios? How much to they affect maternal health and welfare?
